# *The Organon* and Materia Medica Club of the Bay Cities of California

**Published:** 1896-08

**Authors:** W. E. Ledyard

**Affiliations:** Secretary


### *THE ORGANON* AND MATERIA MEDICA CLUB OF THE BAY CITIES OF CALIFORNIA.

The regular semi-monthly meeting was held at the office of Dr. J. M. Selfridge, in Oakland, Friday evening, March 6th, 1896.

Members present : Drs. J. M. Selfridge, A. McNeil, W. E. Ledyard, M. T. Wilson, G. J. Augur, M. F. Underwood, and C. M. Selfridge.

The meeting was called to order at 8.20 o'clock by the President, Dr. J. M. Selfridge. The minutes of the previous meeting were read by the Secretary, and, after corrections by Drs. J. M. Selfridge and McNeil, were approved.

Dr. McNeil then said he would like to say a few words in regard to Swanism. He said : There has been a tendency for some of our school to follow the errors of Swan. I accuse

Swan of three errors: 1. He uses unproved remedies. 2. He gives drugs for the names of diseases. 3. He compounds drugs. For instance, what he calls "Bellpul" is an unproved remedy and is a combination of several remedies. What he calls "Tissue Twelve Crude" has twelve drugs combined and it is entirely unproved. He has never given provings of these combinations to the profession. The three errors I have just mentioned are the three differences of opinion between Samuel Swan and Samuel Hahnemann, and we cannot serve two masters. If we prescribe according to Swan's teaching we do not practice Homœopathy.

Dr. J. M. Selfridge—I do not condemn Swan entirely, but neither do I approve of combined remedies unless proven. I must admit, however, that splendid remedies have been given to the profession in this way. I have used Swan's *Tuberculinum* without considering whether it fully corresponded with the guiding symptoms, and had splendid results.

Dr. Augur—Even in giving the proven remedies we do not always stop to analyze all of the symptoms, as, for instance, in epidemic remedies. If the symptoms were summed up in such cases perhaps they would not all correspond with the guiding symptoms of the remedies given.

Dr. J. M. Selfridge—I will cite a case. Miss L——, aged sixteen, brown hair and eyes, tall, emaciated, pale, feeble-appearing girl, was brought to my office February 19th, 1896, by her mother who gave the following history: She has always been a delicate child; never could endure anything, and during the last few years has grown very rapidly, and for three months past has become rapidly weak and lifeless. Never ate or slept well; has had frequent attacks of diarrhœa; could not hold her urine; has cold feet and dry, hot hands day and night. Has never menstruated. Was under the care of a homœopath for about a year and an allopath for about three months, each of whom concluded that there was something wrong with the womb or the orifices of the body, and the usual examination was urged, which was refused. Further inquiry elicited the fact that the mother had lost four sisters and three brothers with consumption, while her father died of cancer of the stomach, her mother suffered with rheumatism, and was found dead in her bed from, probably, heart disease. The patient herself had not been troubled with a cough. A careful examination of the lungs by the most approved tests did not discover the presence of tubercle in the lungs. Diagnosis: phthisical cachexia—with a tendency to the deposit of tubercle in the mesenteric glands. I prescribed Swan's *Tuberculinum*$^{dmm}$, one powder. A generous diet, out-door exercise were also recommended, the exercise not to be continued to fatigue, and being careful not to take cold. Result: prompt and continuous improvement from the first. March 4th, just fourteen days from the only dose of medicine, she reports: no diarrhœa from the first dose; bowels regular; appetite good; sleeps well; no cold feet or hot hands; strength improving daily; can run uphill with but little fatigue; feels sprightly and well; cheeks filling out and begin to have a rosy hue.

Dr. McNeil—I congratulate the doctor on making what I consider an accidental cure. *Tuberculinum* is a drug for tuberculosis, but it is not *the* drug. In that case you did not get the true pathogenesis of the drug, and this is not the way to prescribe homœopathically. Because you gave the DMM potency does not make it Homœopathy.

Dr. J. M. Selfridge—I reported another case some time ago of a woman with a sub-peritoneal tumor in the womb. I gave her *Tuberculinum*$^{dmm}$, and in a few months the tumor was gone. I do not hold that this is an example that every one should go by, but we do sometimes get results in this way.

Dr. Augur—Even when we prescribe from key-note symptoms we do not always get results.

Dr. McNeil—I think you will all acknowledge that if we can cure consumption with Calcarea, Phosphorus, and many other drugs, that *Tuberculinum* is not indicated in all cases. We need clinical provings to add to our other provings.

Dr. J. M. Selfridge—I am not an apologist for Swan. In the case I reported there were no sputa or cough, but simply the lineage to direct me. In the case of tumor there was a deposit of tubercle in the lungs. Swan's idea is that a case of consumption is a proving of *Tuberculinum.*

Dr. Ledyard—From what I have read and heard of these nosodes, I judge that the correct use of them is that after you have made a close study of the case, and fail to find symptoms enough for the indicated remedy, you give a dose of the nosode to clear up the case.

Dr. J. M. Selfridge—A great many of the symptoms given in the *Guiding Symptoms* are the ones I have given in my case. Symptoms observed on the sick only.

Dr. Underwood—Case, little girl very delicate. I took the case very carefully, but could not decide upon a remedy at all. I gave *Tuberculinum*$^{cm}$, one dose, and from a puny, delicate girl of sixteen she has developed into a rosy, buxom girl.

Dr. McNeil—Bœnninghausen said that nosodes were not indicated in recent cases. A nosode should not be given for a recent case of itch, but if the itch had been suppressed then it could be given. We must not give them for the name of the disease, but for the totality of the symptoms.

Dr. J. M. Selfridge—I agree with you on that point.

Dr. Selfridge then read from *The Organon,* Sections 71 and 72.

### Discussion.

Dr. Wilson—I believe that when we were discussing these sections before, the question of the self-limitation of disease was brought up. There are many cases which might be called self-limited, but if this idea is allowed to be accepted, will not the question arise as to whether our remedies or the self-limitation of the disease has been the cure? If this is accepted, I think it will put us all at sea as regards the cure of disease.

Dr. J. M. Selfridge—Some diseases do run their own course, as measles, etc., and this is generally accepted as a fact.

Dr. Wilson—Enteric diseases, such as colic, we usually cure in a short time, but if this idea of self-limitation is accepted we might believe that such cases often get well of themselves.

Dr. McNeil—Hahnemann lays down very clearly the diseases that are self-limiting, and those that are not. It is a duty we owe to ourselves to find out whether the patient was cured by our remedy or got well through the self-limitation of the disease. The acute diseases are self-limiting, but the chronic diseases are not.

Section 73 was read.

### Discussion.

Dr. McNeil—Rademacher first brought up the question of epidemic diseases, and later it was enlarged upon by Grauvogl. Speaking from a therapeutic standpoint, I think that epidemic diseases should all be treated alike. In every case in which we would give the epidemic remedy it would be given according to homœopathic principles. A great many diseases diagnostically we must call different diseases, but if we examine them closely we will find them to be very similar. The epidemic remedy cures these diseases because it is homœopathic to them.

Dr. Augur—Dr. McNeil states that in the epidemic disease there is a type that may not be recognizable at first, but may be later; and by giving the epidemic remedy for such a condition it will cure. So it is in tuberculosis. There is a common cause for the production of this disease in all cases, the symptoms varying somewhat in each individual case, and yet the doctor says they must not be treated similarly. I fail to understand how he makes this distinction.

Dr. McNeil—Dr. Augur starts in with a fundamental error. Tuberculosis is not from a common cause. Hahnemann does not depend on one remedy for psora. The different symptoms that will appear in consumption of the lungs—for instance, as cough of all kinds, different kinds of expectoration, etc.—will readily tell of the different remedies indicated.

Dr. J. M. Selfridge—I do not think that Dr. Augur thinks that *Tuberculinum* would cure every case of consumption.

Dr. Augur—Not at all.

Dr. Ledyard—It appears to me that it is prescribing on pathological grounds.

Dr. J. M. Selfridge—In the case I cited the patient had symptoms that are produced by tubercle. Tubercle injected into animals will cause symptoms of tuberculosis.

Dr. Ledyard—I think there must be a predisposition to tuberculosis.

The meeting was then declared adjourned, to meet again the third Friday in March at the office of Dr. George H. Martin in San Francisco, when the reading of *The Organon* would be commenced at Section 74.

W. E. LEDYARD, *Secretary.*

Reported by Eleanor F. Martin, M. D.

---

